# Bis(nitrato-κ*O*)(3-oxapentane-1,5-diamine-κ^3^ *N*,*O*,*N*′)zinc(II)

**DOI:** 10.1107/S1600536811018010

**Published:** 2011-05-20

**Authors:** Fei Jia, Fan Kou, Bin Liu, Bei-Bei Jia, Hui-Lu Wu

**Affiliations:** a School of Chemical and Biological Engineering, Lanzhou Jiaotong University, Lanzhou 730070, People’s Republic of China

## Abstract

In the title compound, [Zn(NO_3_)_2_(C_4_H_12_N_2_O)], the Zn^II^ atom is *N*,*O*,*N*′-chelated by a 3-oxapentane-1,5-diamine ligand and is further coordinated by two nitrate anions in a distorted trigonal–bipyramidal geometry. Inter­molecular N—H⋯O hydrogen bonding is present in the crystal structure. A short O⋯O contact of 2.816 (8) Å is observed between the nitrate anions of adjacent mol­ecules.

## Related literature

For polydentate amine ligands in metal complexes, see: Fanshawe *et al.* (2000 ▶). For applications of metal complexes with a tridentate amine ligand, see: Junk & Steed (2007 ▶). For a description of the geometry of complexes with five-coordinate metal atoms, see: Addison *et al.* (1984 ▶).
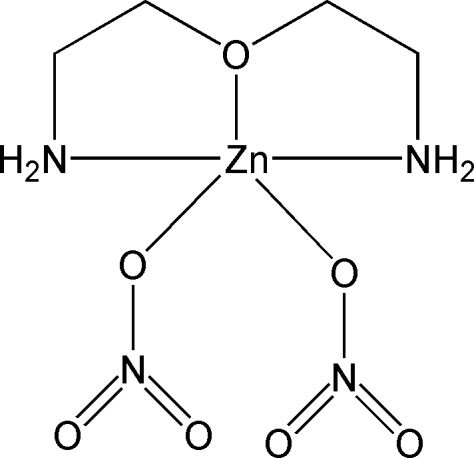


## Experimental

### 

#### Crystal data

[Zn(NO_3_)_2_(C_4_H_12_N_2_O)]
*M* *_r_* = 293.55
Triclinic, 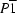

*a* = 8.031 (19) Å
*b* = 8.034 (19) Å
*c* = 9.55 (2) Å
α = 103.97 (2)°
β = 101.90 (2)°
γ = 115.879 (18)°
*V* = 503 (2) Å^3^
*Z* = 2
Mo *K*α radiation
μ = 2.48 mm^−1^
*T* = 296 K
0.30 × 0.28 × 0.26 mm

#### Data collection

Bruker SMART 1000 diffractometer
Absorption correction: multi-scan (*SADABS*; Sheldrick, 2001 ▶) *T*_min_ = 0.524, *T*_max_ = 0.565
2969 measured reflections
1712 independent reflections
1543 reflections with *I* > 2σ(*I*)
*R*_int_ = 0.016

#### Refinement

*R*[*F* ^2^ > 2σ(*F* ^2^)] = 0.026
*wR*(*F* ^2^) = 0.065
*S* = 1.08
1712 reflections
145 parameters
H-atom parameters constrained
Δρ_max_ = 0.39 e Å^−3^
Δρ_min_ = −0.30 e Å^−3^

### 

Data collection: *SMART* (Bruker, 2001 ▶); cell refinement: *SAINT* (Bruker, 2001 ▶); data reduction: *SAINT*; program(s) used to solve structure: *SHELXTL* (Sheldrick, 2008 ▶); program(s) used to refine structure: *SHELXTL*; molecular graphics: *SHELXTL*; software used to prepare material for publication: *SHELXTL*.

## Supplementary Material

Crystal structure: contains datablocks global, I. DOI: 10.1107/S1600536811018010/xu5211sup1.cif

Structure factors: contains datablocks I. DOI: 10.1107/S1600536811018010/xu5211Isup2.hkl

Additional supplementary materials:  crystallographic information; 3D view; checkCIF report

## Figures and Tables

**Table 1:** Hydrogen-bond geometry (Å, °)

*D*—H⋯*A*	*D*—H	H⋯*A*	*D*⋯*A*	*D*—H⋯*A*
N1—H3*A*⋯O2^i^	0.90	2.39	3.217 (7)	152
N1—H3*B*⋯O6^ii^	0.90	2.51	3.168 (9)	130
N2—H2*A*⋯O4^iii^	0.90	2.42	3.058 (8)	128
N2—H2*B*⋯O5^iv^	0.90	2.41	3.201 (9)	145
N2—H2*B*⋯O3^iv^	0.90	2.49	3.106 (6)	126

Symmetry codes: (i) 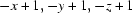; (ii) 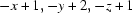; (iii) 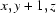; (iv) 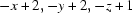.

**Fig. 1.**
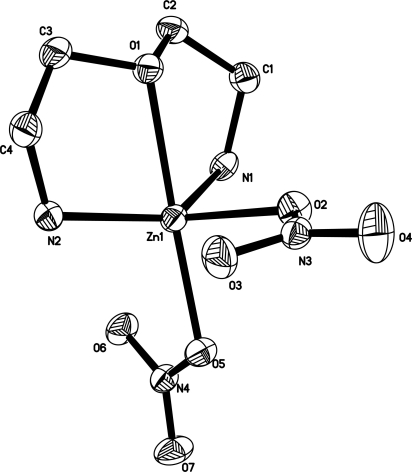
The molecular structure of the title compound. Displacement ellipsoids are drawn at the 30% probability level. H atoms have been omitted for clarity.

## supplementary crystallographic information

### Comment

Polydentate amine ligands generally coordinate to transition metal ions using all of the available nitrogen atoms as donors (Fanshawe *et al.*, 2000). Transition metal coordination complexes involving tridentate amines as ligands have attracted solid attention for their role as model compounds for bioinorganic systems, as building blocks in supramolecular assemblies and as catalysts (Junk & Steed, 2007). 3-oxapentane-1,5-diamine as the one of the classics of polyamines behaves as a tridentate ligand that can form three coordinative bonds with a metal atom through the long pair electrons on two N atoms and one oxygen.

The Zn^II^ in the complex is N,O,N'-chelated by a 3-oxapentane-1,5-diamine ligand and is further coordinated by two nitrate anions. The coordination geometry of the Zn^II^ ion may be best described as distorted trigonal bipyramidal (tau = 2/3). The parameter tau is defined as (beta-alpha)/60) [where beta= O(5)–Zn(1)–O(1), alpha = N(2)–Zn(1)–N(1)] and its value varies from 0 (in regular square-base pyramidal) to 1 (in regular trigonal bipyramidal) (Addison *et al.*, 1984). The equatorial plane is occupied by two N atoms of the ligand, and one O atom of nitrate anions, whereas the Zn^II^ ion protrudes towards O5 by 0.337 Å from the plane of atoms N1/N2/O2. The axial positions are occupied by O1 and O5 atoms. The crystal structure is mainly stabilized by the intermolecular H–bond.

### Experimental

To a stirred solution of 3-oxapentane-1,5-diamine(0.104 g, 0.10 mmol) in EtOH (10 ml) was added Zn(NO_3)_2(H_2_O)_6_(0.297 g 0.1 mmol) in EtOH (5 ml).A White crystalline product formed rapidly.The precipitate was filtered off, wash with EtOH and *in vacuo*.The dried precipitate was dissolved in DMF resulting in a colourless solutoin.The crystals suitable for X-ray diffraction studies were obtained by ether diffusion into DMF after several days at room temperature.Yield,0.115 g(28%).(found:*C*,16.45;H,3.99;N,19.43.Calcd.: C, 16.37; H, 4.12; N, 19.09).

### Refinement

H atoms were placed in calculated positiosn with C—H = 0.97 and N—H = 0.90 Å, and refined in a riding-model approximation with *U*_iso_(H) = 1.2*U*_eq_(C,N).

### Crystal data

**Table:**

[Zn(NO_3_)_2_(C_4_H_12_N_2_O)]	*Z* = 2
*M**_r_* = 293.55	*F*(000) = 300
Triclinic, *P*1	*D*_x_ = 1.940 Mg m^−^^3^
Hall symbol: -P 1	Mo *K*α radiation, λ = 0.71073 Å
*a* = 8.031 (19) Å	Cell parameters from 1744 reflections
*b* = 8.034 (19) Å	θ = 2.4–26.2°
*c* = 9.55 (2) Å	µ = 2.48 mm^−^^1^
α = 103.97 (2)°	*T* = 296 K
β = 101.90 (2)°	Block, colourless
γ = 115.879 (18)°	0.30 × 0.28 × 0.26 mm
*V* = 503 (2) Å^3^	

### Data collection

**Table:**

Bruker SMART 1000 diffractometer	1712 independent reflections
Radiation source: fine-focus sealed tube	1543 reflections with *I* > 2σ(*I*)
graphite	*R*_int_ = 0.016
ω scans	θ_max_ = 25.0°, θ_min_ = 2.4°
Absorption correction: multi-scan (*SADABS*; Sheldrick, 2001)	*h* = −9→9
*T*_min_ = 0.524, *T*_max_ = 0.565	*k* = −9→9
2969 measured reflections	*l* = −11→10

### Refinement

**Table:**

Refinement on *F*^2^	Primary atom site location: structure-invariant direct methods
Least-squares matrix: full	Secondary atom site location: difference Fourier map
*R*[*F*^2^ > 2σ(*F*^2^)] = 0.026	Hydrogen site location: inferred from neighbouring sites
*wR*(*F*^2^) = 0.065	H-atom parameters constrained
*S* = 1.08	*w* = 1/[σ^2^(*F*_o_^2^) + (0.0269*P*)^2^ + 0.3287*P*] where *P* = (*F*_o_^2^ + 2*F*_c_^2^)/3
1712 reflections	(Δ/σ)_max_ < 0.001
145 parameters	Δρ_max_ = 0.39 e Å^−^^3^
0 restraints	Δρ_min_ = −0.30 e Å^−^^3^

### Special details

**Table:**

Geometry. All e.s.d.'s (except the e.s.d. in the dihedral angle between two l.s. planes) are estimated using the full covariance matrix. The cell e.s.d.'s are taken into account individually in the estimation of e.s.d.'s in distances, angles and torsion angles; correlations between e.s.d.'s in cell parameters are only used when they are defined by crystal symmetry. An approximate (isotropic) treatment of cell e.s.d.'s is used for estimating e.s.d.'s involving l.s. planes.
Refinement. Refinement of *F*^2^ against ALL reflections. The weighted *R*-factor *wR* and goodness of fit *S* are based on *F*^2^, conventional *R*-factors *R* are based on *F*, with *F* set to zero for negative *F*^2^. The threshold expression of *F*^2^ > σ(*F*^2^) is used only for calculating *R*-factors(gt) *etc*. and is not relevant to the choice of reflections for refinement. *R*-factors based on *F*^2^ are statistically about twice as large as those based on *F*, and *R*- factors based on ALL data will be even larger.

### Fractional atomic coordinates and isotropic or equivalent isotropic displacement parameters (Å^2^)

**Table:**

	*x*	*y*	*z*	*U*_iso_*/*U*_eq_	
C1	0.1984 (4)	0.4429 (4)	0.1842 (4)	0.0421 (7)	
H4A	0.0585	0.3840	0.1683	0.050*	
H4B	0.2276	0.3362	0.1631	0.050*	
C2	0.2448 (4)	0.5479 (4)	0.0773 (3)	0.0408 (7)	
H3C	0.1756	0.4536	−0.0289	0.049*	
H3D	0.2044	0.6464	0.0907	0.049*	
C3	0.5347 (5)	0.8083 (5)	0.0668 (4)	0.0447 (8)	
H1A	0.4661	0.8816	0.0797	0.054*	
H1B	0.5209	0.7623	−0.0409	0.054*	
C4	0.7451 (5)	0.9362 (5)	0.1657 (4)	0.0443 (8)	
H2C	0.8153	0.8670	0.1428	0.053*	
H2D	0.8031	1.0575	0.1449	0.053*	
N1	0.3161 (3)	0.5833 (4)	0.3447 (3)	0.0354 (5)	
H3A	0.3025	0.5149	0.4077	0.043*	
H3B	0.2702	0.6660	0.3699	0.043*	
N2	0.7660 (3)	0.9873 (3)	0.3291 (3)	0.0350 (5)	
H2A	0.7262	1.0754	0.3551	0.042*	
H2B	0.8947	1.0466	0.3874	0.042*	
N3	0.8109 (4)	0.5303 (3)	0.3367 (3)	0.0344 (5)	
N4	0.7273 (4)	0.9449 (3)	0.6934 (3)	0.0351 (6)	
O1	0.4541 (3)	0.6427 (3)	0.1137 (2)	0.0370 (5)	
O2	0.6435 (3)	0.5019 (3)	0.3413 (2)	0.0425 (5)	
O3	0.9375 (3)	0.6943 (3)	0.3458 (3)	0.0538 (6)	
O4	0.8395 (4)	0.3919 (4)	0.3230 (4)	0.0700 (8)	
O5	0.7711 (3)	0.8330 (3)	0.6081 (2)	0.0367 (5)	
O6	0.6094 (3)	0.9873 (3)	0.6285 (3)	0.0489 (6)	
O7	0.8036 (4)	1.0070 (4)	0.8329 (2)	0.0556 (6)	
Zn1	0.60715 (5)	0.74633 (5)	0.37628 (4)	0.03129 (12)	

### Atomic displacement parameters (Å^2^)

**Table:**

	*U*^11^	*U*^22^	*U*^33^	*U*^12^	*U*^13^	*U*^23^
C1	0.0341 (16)	0.0388 (16)	0.0448 (18)	0.0122 (13)	0.0134 (14)	0.0155 (14)
C2	0.0346 (16)	0.0391 (16)	0.0352 (16)	0.0135 (13)	0.0052 (13)	0.0097 (13)
C3	0.0485 (19)	0.0457 (17)	0.0352 (17)	0.0162 (15)	0.0176 (15)	0.0209 (14)
C4	0.0460 (18)	0.0410 (17)	0.0428 (18)	0.0156 (14)	0.0213 (15)	0.0196 (14)
N1	0.0376 (13)	0.0451 (14)	0.0364 (13)	0.0247 (12)	0.0195 (11)	0.0232 (11)
N2	0.0336 (13)	0.0318 (12)	0.0356 (13)	0.0148 (10)	0.0092 (11)	0.0129 (10)
N3	0.0433 (14)	0.0363 (13)	0.0311 (13)	0.0245 (12)	0.0157 (11)	0.0145 (10)
N4	0.0397 (14)	0.0386 (13)	0.0337 (14)	0.0239 (12)	0.0139 (11)	0.0162 (11)
O1	0.0351 (11)	0.0374 (10)	0.0339 (11)	0.0131 (9)	0.0137 (9)	0.0159 (9)
O2	0.0388 (12)	0.0442 (12)	0.0500 (13)	0.0251 (10)	0.0185 (10)	0.0165 (10)
O3	0.0541 (14)	0.0401 (12)	0.0583 (15)	0.0136 (11)	0.0231 (12)	0.0227 (11)
O4	0.0750 (18)	0.0592 (15)	0.114 (2)	0.0528 (15)	0.0505 (17)	0.0426 (16)
O5	0.0444 (12)	0.0435 (11)	0.0305 (10)	0.0298 (10)	0.0135 (9)	0.0123 (9)
O6	0.0525 (13)	0.0560 (13)	0.0527 (14)	0.0402 (12)	0.0129 (11)	0.0237 (11)
O7	0.0754 (17)	0.0698 (15)	0.0279 (12)	0.0482 (14)	0.0122 (11)	0.0121 (11)
Zn1	0.03247 (19)	0.03232 (19)	0.03040 (19)	0.01648 (14)	0.01121 (14)	0.01408 (14)

### Geometric parameters (Å, °)

**Table:**

C1—N1	1.467 (4)	N1—H3A	0.9000
C1—C2	1.480 (5)	N1—H3B	0.9000
C1—H4A	0.9700	N2—Zn1	2.020 (4)
C1—H4B	0.9700	N2—H2A	0.9000
C2—O1	1.429 (5)	N2—H2B	0.9000
C2—H3C	0.9700	N3—O4	1.214 (4)
C2—H3D	0.9700	N3—O3	1.231 (4)
C3—O1	1.429 (4)	N3—O2	1.273 (4)
C3—C4	1.470 (5)	N4—O7	1.215 (4)
C3—H1A	0.9700	N4—O6	1.240 (3)
C3—H1B	0.9700	N4—O5	1.279 (3)
C4—N2	1.468 (5)	O1—Zn1	2.307 (5)
C4—H2C	0.9700	O2—Zn1	2.068 (5)
C4—H2D	0.9700	O5—Zn1	2.091 (4)
N1—Zn1	2.029 (5)		
			
N1—C1—C2	110.1 (3)	H3A—N1—H3B	108.0
N1—C1—H4A	109.6	C4—N2—Zn1	112.71 (19)
C2—C1—H4A	109.6	C4—N2—H2A	109.1
N1—C1—H4B	109.6	Zn1—N2—H2A	109.1
C2—C1—H4B	109.6	C4—N2—H2B	109.1
H4A—C1—H4B	108.2	Zn1—N2—H2B	109.1
O1—C2—C1	106.9 (2)	H2A—N2—H2B	107.8
O1—C2—H3C	110.3	O4—N3—O3	122.0 (3)
C1—C2—H3C	110.3	O4—N3—O2	117.9 (3)
O1—C2—H3D	110.3	O3—N3—O2	120.0 (3)
C1—C2—H3D	110.3	O7—N4—O6	122.9 (3)
H3C—C2—H3D	108.6	O7—N4—O5	119.3 (2)
O1—C3—C4	106.7 (3)	O6—N4—O5	117.8 (3)
O1—C3—H1A	110.4	C2—O1—C3	114.1 (2)
C4—C3—H1A	110.4	C2—O1—Zn1	108.81 (17)
O1—C3—H1B	110.4	C3—O1—Zn1	109.59 (19)
C4—C3—H1B	110.4	N3—O2—Zn1	116.47 (18)
H1A—C3—H1B	108.6	N4—O5—Zn1	109.2 (2)
N2—C4—C3	110.2 (3)	N2—Zn1—N1	133.42 (10)
N2—C4—H2C	109.6	N2—Zn1—O2	124.63 (18)
C3—C4—H2C	109.6	N1—Zn1—O2	93.24 (17)
N2—C4—H2D	109.6	N2—Zn1—O5	102.15 (13)
C3—C4—H2D	109.6	N1—Zn1—O5	107.77 (13)
H2C—C4—H2D	108.1	O2—Zn1—O5	84.95 (10)
C1—N1—Zn1	111.6 (2)	N2—Zn1—O1	76.61 (12)
C1—N1—H3A	109.3	N1—Zn1—O1	77.29 (11)
Zn1—N1—H3A	109.3	O2—Zn1—O1	90.28 (10)
C1—N1—H3B	109.3	O5—Zn1—O1	173.20 (8)
Zn1—N1—H3B	109.3		
			
N1—C1—C2—O1	55.3 (3)	C1—N1—Zn1—O5	−153.7 (2)
O1—C3—C4—N2	−53.8 (3)	C1—N1—Zn1—O1	21.62 (19)
C2—C1—N1—Zn1	−49.5 (3)	N3—O2—Zn1—N2	22.7 (2)
C3—C4—N2—Zn1	48.8 (3)	N3—O2—Zn1—N1	173.84 (19)
C1—C2—O1—C3	−157.2 (2)	N3—O2—Zn1—O5	−78.6 (2)
C1—C2—O1—Zn1	−34.5 (3)	N3—O2—Zn1—O1	96.6 (2)
C4—C3—O1—C2	156.3 (3)	N4—O5—Zn1—N2	81.0 (3)
C4—C3—O1—Zn1	34.0 (3)	N4—O5—Zn1—N1	−62.8 (2)
O4—N3—O2—Zn1	176.9 (2)	N4—O5—Zn1—O2	−154.59 (18)
O3—N3—O2—Zn1	−3.2 (3)	N4—O5—Zn1—O1	159.8 (5)
O7—N4—O5—Zn1	178.1 (2)	C2—O1—Zn1—N2	−133.23 (19)
O6—N4—O5—Zn1	−2.8 (3)	C3—O1—Zn1—N2	−7.84 (19)
C4—N2—Zn1—N1	−78.7 (2)	C2—O1—Zn1—N1	7.84 (17)
C4—N2—Zn1—O2	59.7 (2)	C3—O1—Zn1—N1	133.2 (2)
C4—N2—Zn1—O5	152.0 (2)	C2—O1—Zn1—O2	101.1 (2)
C4—N2—Zn1—O1	−21.2 (2)	C3—O1—Zn1—O2	−133.5 (2)
C1—N1—Zn1—N2	78.9 (3)	C2—O1—Zn1—O5	146.5 (6)
C1—N1—Zn1—O2	−67.9 (2)	C3—O1—Zn1—O5	−88.1 (7)

### Hydrogen-bond geometry (Å, °)

**Table:**

*D*—H···*A*	*D*—H	H···*A*	*D*···*A*	*D*—H···*A*
N1—H3A···O2^i^	0.90	2.39	3.217 (7)	152
N1—H3B···O6^ii^	0.90	2.51	3.168 (9)	130
N2—H2A···O4^iii^	0.90	2.42	3.058 (8)	128
N2—H2B···O5^iv^	0.90	2.41	3.201 (9)	145
N2—H2B···O3^iv^	0.90	2.49	3.106 (6)	126

Symmetry codes: (i) −*x*+1, −*y*+1, −*z*+1; (ii) −*x*+1, −*y*+2, −*z*+1; (iii) *x*, *y*+1, *z*; (iv) −*x*+2, −*y*+2, −*z*+1.
